# A threshold level of oxalate oxidase transgene expression reduces *Cryphonectria parasitica*-induced necrosis in a transgenic American chestnut (*Castanea dentata*) leaf bioassay

**DOI:** 10.1007/s11248-013-9708-5

**Published:** 2013-03-31

**Authors:** Bo Zhang, Allison D. Oakes, Andrew E. Newhouse, Kathleen M. Baier, Charles A. Maynard, William A. Powell

**Affiliations:** 1 Department of Environmental and Forest Biology, State University of New York-College of Environmental Science and Forestry, 1 Forestry Dr., Syracuse, NY 13210 USA; 2 Department of Forest and Natural Resources Management, State University of New York-College of Environmental Science and Forestry, 1 Forestry Dr., Syracuse, NY 13210 USA

**Keywords:** Oxalate oxidase (OxO), *Cryphonectria parasitica*, *Castanea dentata*, Transgenic tree

## Abstract

American chestnut (*Castanea dentata*) was transformed with a wheat oxalate oxidase (*oxo*) gene in an effort to degrade the oxalic acid (OA) secreted by the fungus *Cryphonectria parasitica*, thus decreasing its virulence. Expression of OxO was examined under two promoters: a strong constitutive promoter, CaMV 35S, and a predominantly vascular promoter, *VspB*. *Oxo* gene transcription was quantified by RT-qPCR. Relative expression of OxO varied approximately 200 fold among events produced with the 35S-OxO. The lowest 35S-OxO event expressed approximately 3,000 fold higher than the highest *VspB*-OxO event. This was potentially due to the tissue-specific nature of the *VspB*-controlled expression, the strength of the CaMV 35S constitutive promoter, or position effects. Leaf assays measuring necrotic lesion length were conducted to better understand the relationship between OxO expression level and the blight fungus *in planta*. A threshold response was observed between the OxO expression level and the *C. parasitica* lesion length. Five events of the 35S-OxO line showed significantly reduced lesion length compared to the blight-susceptible American chestnut. More importantly, the lesion length in these five events was reduced to the same level as the blight-resistant Chinese chestnut, *C. mollissima*. This is the first report on enhanced pathogen resistance in transgenic American chestnut.

## Introduction

The American chestnut, *Castanea dentata* (Marsh.) Borkh. is a tree of great historical, ecological, and economic significance. To regain this tree’s historical potential, blight resistant trees are needed.

In the late nineteenth century, *Cryphonectria parasitica* (Murrill.) Barr. was unintentionally brought into the United States on nursery stock from Asia (Anagnostakis and Hillman 1992). The fungus was first reported by H. W. Merkel in 1905 at the New York Zoological Garden (1905). Within 50 years, it killed almost all of the estimated 4 billion American chestnut trees in the eastern forests of the United States (Roane et al. 1986). Chestnut blight also severely affects European chestnut (Robin and Heiniger 2001) but is currently being controlled in many areas by hypovirulence. Hypovirulence has only had limited success in the US (Milgroom and Cortesi 2004).

The fungus infects wounded stem tissue, secreting OA to decrease the pH of the infected tissue to levels that are toxic for the tree, but optimal for fungal enzymes. OA is also known to induce programmed cell death in some pathogen/host systems (Errakhi et al. 2008; Kim et al. 2008). Hyphae then spread through the cambium, forming a canker that will eventually kill the tree above the infection site by preventing the transfer of water and nutrients (Griffin 1986; Newhouse 1990). Fortunately for the host, the fungus does not infect chestnut roots, therefore allowing the growth of shoots at the root collar, keeping the tree alive (Anderson 1914). However, this regrowth is only temporary because these sprouts will eventually be re-infected by the fungus and die back to the ground. It is this continuing cycle that reduced American chestnut, once a great canopy tree, to no more than an early-succession-stage shrub today (Ellison et al. 2005).

Efforts are underway to develop blight resistant American chestnuts with the goal of restoring this important species back to its natural range. This study involves a potential resistance-enhancing gene from wheat (*Triticum aestivum*; Lane et al. 1993) called *oxo*. This gene, along with the screenable marker *gfp* (green fluorescent protein, Prasher et al. 1992), NPTII (neomycin phosphotransferase II) and BAR (bialaphos resistance gene, selectable marker genes), was used to transform American chestnut somatic embryos through *Agrobacterium*-mediated co-transformation (Zhang et al. 2011). Gene *oxo* codes for the enzyme oxalate oxidase (OxO) (EC 1.2.3.4), which catalyzes the degradation of OA to carbon dioxide and hydrogen peroxide (Berna and Bernier 1999). Its expression in transgenic chestnut was driven by two promoters: a constitutive *Cauliflower mosaic virus* (CaMV) 35S promoter and a predominantly vascular VspB promoter (Zhang et al. 2011). In *C. parasitica*, oxalate is produced in seven- to 18-fold higher concentrations in virulent strains compared to hypovirulent strains (Havir and Anagnostakis 1983), suggesting it plays an important role in virulence. Oxalate has also been shown to be essential to *C. parasitica*’s ability to form cankers on chestnut (Chen et al. 2010).

In this study, the effect of the OxO on *C. parasitica* was investigated to determine whether there was a particular level of expression at which OxO would reduce the ability of *C. parasitica* to induce necrotic lesions.

## Materials and methods

### Plant material and growth conditions

Somatic embryogenic clumps of wild-type American chestnut “Ellis 1” and “WB 275-27”, induced from zygotic embryos, were used as target material in transformation experiments. Induction of the embryogenic process, proliferation, maintenance of the embryogenic capacity, maturation, germination and plantlet conversion steps were carried out according to Polin et al. (2006). In addition, axillary shoot cultures of the American and Chinese chestnut lines Ellis #1 and Qing 3 (from The American Chestnut Foundation), respectively, were established in vitro. Somatic embryo cultures were grown in the dark and axillary shoot cultures were grown under a 16-h photoperiod at a light intensity of 45–60 μmol/m^2^/s at 25 ± 2 °C. Regenerated plants were fertilized with 300 mg/L Miracid^®^ (Miracle-Gro^®^, The Scotts Company LLC, Marysville, OH, USA) two or three times a week both in the growth chamber and the greenhouse.

### Co-transformation and regeneration

The co-transformation procedure was developed based on the transformation protocol described by Polin et al. (2006). Vectors p35S-OxO, pTACF3 (*VspB*-OxO), pWVK147 (empty control vector with the same backbone as p35S-OxO and pTACF3, but without the promoter-gene cassette) and pGFP (contains the Green Fluorescent Protein gene under a 35S promoter) were introduced into *Agrobacterium tumefaciens* (strain EHA105) separately via electroporation so that each *Agrobacterium* had only one of the vectors. Typically 8–10 weeks were required to isolate a GFP-positive embryo cluster for PCR confirmation and regeneration. Only PCR-confirmed transgenic events were regenerated into whole plants. Both Ellis 1 (non-transformed American chestnut) and Qing 3 (Chinese chestnut) were regenerated in vitro. Four events were generated in the VspB-OxO line, 22 in the 35S-OxO line, four in the empty vector line, and six in the GFP only line.

After 28–36 weeks the American chestnut embryos regenerated into shoots, which were multiplied through axillary branching, and rooted. Rooting took an additional 8 weeks to produce plantlets ready for acclimatization. Plants were grown for 3 months in the growth chamber before being moved to the greenhouse. A total of 12–14 months were required to produce a potted American chestnut plant in the greenhouse from the somatic embryos.

### OxO expression level quantification

All four events in the VspB-OxO line, 17 events in the 35S-OxO line, two events in the empty vector line, and two events in the GFP-only line were assayed. Total RNA was extracted from stem tissues (either tissue culture shoots or small young stems from potted plants). For tissue culture shoots, four to six shoots were used per event and total RNA was pooled before cDNA synthesis. Three to four stems from different potted plants from each event were used for RNA extraction; again, RNA from different individuals within an event was pooled before cDNA synthesis. Total RNA was extracted using the CTAB method (Chang et al. 1993; Gambino et al. 2008) followed by quantification with a NanoDrop^®^ ND-1000 Spectrophotometer (Thermo Scientific^®^). cDNA was synthesized from ~0.5 μg total RNA using a Qiagen QuantiTect Reverse Transcription Kit (with the optional DNase treatment step included), and diluted cDNA samples (1:5) were used as templates. Each 10 μL qPCR reaction contained 5 μL iQ SYBR Green supermix (Bio-Rad Laboratories, Inc., Hercules, CA, USA), 0.5 μL of each 10 μM primer, 1 μL nuclease-free H_2_O supplied with the SYBR kit, and 3 μL diluted cDNA. Each qPCR reaction was run in triplicate. No-template and non-transgenic samples were used as controls. A two-step qPCR (95° for 3 min, 40 cycles of 95° for 10 s and 60° for 30 s, followed by a melt curve from 61° to 95° at an increment of 1° per 5 s) was performed on a 48-well MiniOpticon Real-Time PCR System (Bio-Rad Laboratories, Inc.) with data analysis using CFX ManagerTM software (Bio-Rad Laboratories, Inc.). The same aliquots of diluted cDNA samples were used for all primer sets in all qPCR runs. Glyceraldehyde 3-phosphate dehydrogenase (GAPDH) was used as the reference gene. Primers used in the qPCR included the *oxo* gene forward (5′ CAG CGG CAA ACT TGG ACT TGA GAA) and reverse (5′ TGC ACT TCC AGT TCA ACG TCG GTA) and the *GAPDH* gene forward (5′ GCT GCA CTA CCA ATT GTC TTG) and reverse (5′ TCA TTG AAG GAC CAT CGA CAG). All primers were synthesized by Sigma-Aldrich^®^.

Oxalate oxidase enzyme activity was confirmed using a leaf staining assays as previously described (Liang et al. 2001). Non-transformed Ellis 1 was used as a control. Assays were performed with and without the OA substrate to ensure the reaction was caused by oxalate oxidase and not another hydrogen peroxide generating enzyme.

### Leaf assay

Leaf assays (Fig. 3b) were conducted using *C. parasitica* strain SG2-3 culture plugs as inoculum. Strain SG2-3 is of medium virulence and has been shown to produce significantly different lesion lengths between leaves of Chinese and American chestnuts (Andrew Newhouse, unpublished). SG2-3 was sub-cultured on Potato Dextrose Agar 3–4 days prior to use. Plugs of SG2-3 were formed just before inoculation with a sterile #1 cork borer (~3 mm diameter) around the perimeter of actively growing *C. parasitica* cultures. Fully-expanded small young leaves (~6–9 cm) from potted chestnut plants were used. One 5 mm wound was made along the midvein on the abaxial surface of each leaf with a #11 blade followed by inoculation with a SG2-3 plug. The wound was approximately 15–20 mm away from the petiole, and less than half the depth of the midvein. The SG2-3 plug was placed directly on top of the wound site with the culture side contacting the wound. Inoculated leaves were placed inoculum-side-up on damp paper towels inside an air-tight plastic dish. The dishes were covered with aluminium foil to keep them dark, and incubated at room temperature (25 ± 2 °C). After 3 days, lesion length was measured along the midvein on the upper side of the leaf, ensuring that the measurements were made on the necrosis, not the initial wound. This procedure was repeated with a highly virulent strain of *C. parasitica*, EP155 (ATCC #38755), on two events and controls. Both SG2-3 and EP155 are typically used by The American Chestnut Foundation (Ashville, NC, USA) to determine levels of blight resistance.

The blight-resistant Chinese chestnut (Qing 3) and the blight-susceptible American chestnut (Ellis #1) were used as non-transgenic controls and standards for blight susceptibility. Two transgenic control lines were used: the GFP-only line and the empty vector pWVK147 control line, along with the *VspB*-OxO line and the 35S-OxO line. Vector pWVK147 has exactly the same vector backbone as those in pTACF3 and p35S-OxO, but without the promoter-*oxo* gene cassette. Six to thirteen leaves were inoculated for each event and the non-transgenic controls, and the mean lesion lengths were compared to both Chinese and American control lines. ANOVA and a series of *t* tests were used to determine significant differences. All plants used were generated from tissue culture (including non-transgenic controls).

## Results

### OxO expression in the *VspB*-OxO and the 35S-OxO transgenic lines

**Fig. 1:**
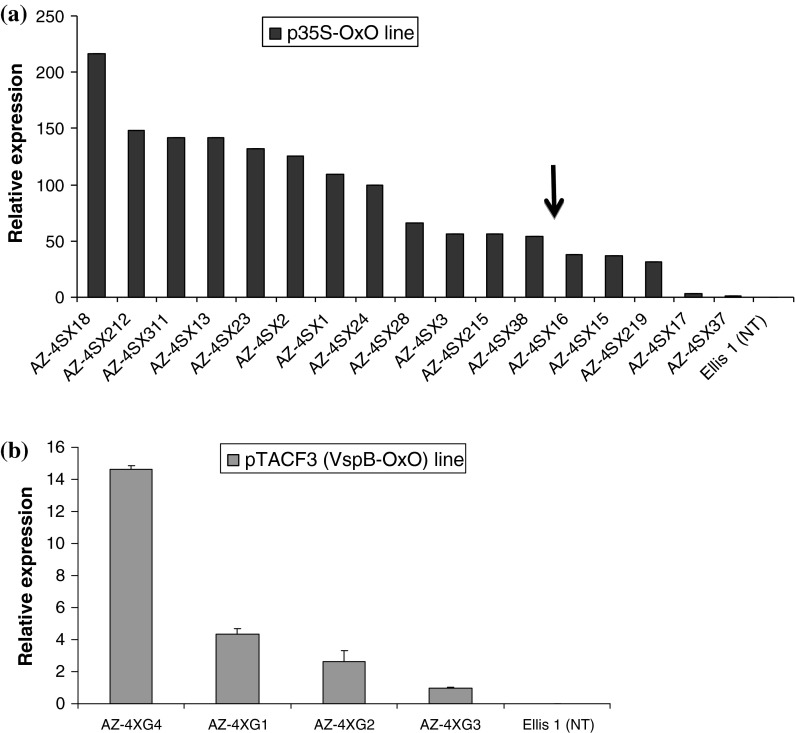
Relative expression levels of the *oxo* gene in tissue culture shoots using RT-qPCR. Non-transgenic (NT) American chestnut controls were the clonal line, Ellis 1, in both graphs. **a** Seventeen transgenic events made with the vector p35S-OxO (containing a constitutive promoter); **b** four transgenic events made with the vector pTACF3 (containing a vascular promoter). GAPDH was used as the reference gene. Data represent the mean of three technical replicates. *Bars* indicate standard error of the mean [note in (**a**) they are present but too small to see]. *Arrow* indicates threshold OxO expression level linked to significant reduction in necrosis (Fig. 3)

Relative OxO transcript levels were quantified by RT-qPCR (Reserve Transcription-quantitative PCR) in both *VspB*-OxO and 35S-OxO transgenic lines using total RNA extracted from tissue culture shoots. OxO expression levels varied widely among the sixteen 35S-OxO events (Fig. 1a) with the highest event, 4SX18, expressing more than 200-fold higher than the lowest event, 4SX37. Within the *VspB*-OxO line, the variation in expression levels was not as great as that in the 35S-OxO line (Fig. 1b), with only about a 14 fold difference between highest to lowest. Moreover, the lowest expressed event in the 35S-OxO line (4SX37) expressed the *oxo* gene more than 3,000-fold higher than the highest expressed event (4XG4) in the *VspB*-OxO line. No OxO expression was observed in the non-transgenic (NT) American chestnut control line Ellis 1 (Fig. 1a, b).

### OxO expression level in tissue culture shoots and stems of potted young plants

**Fig. 2:**
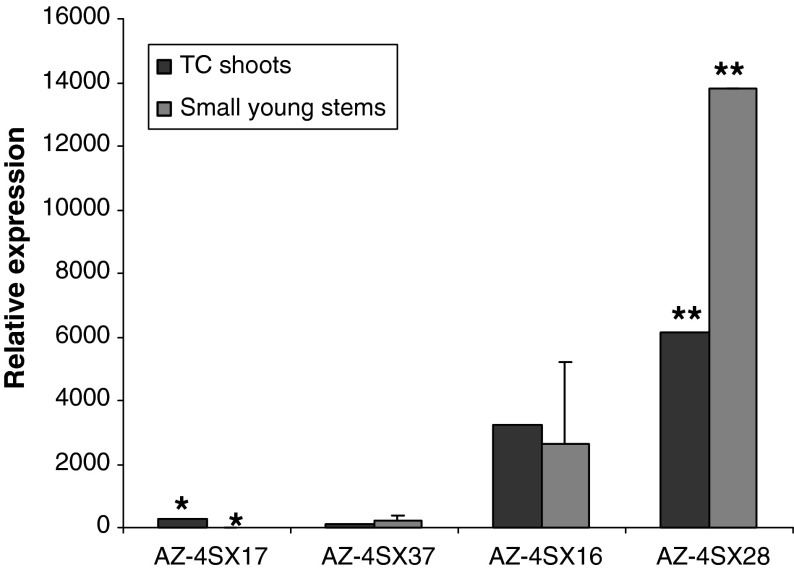
A comparison of OxO expression level in tissue culture shoots (TC shoots, *black column*) and stems (*gray column*) of young potted plants in the greenhouse of the transgenic line p35S-OxO (events AZ-4SX16, 17, 28, and 37). RT-qPCR with GAPDH as the reference gene was used to determine the expression of OxO. *Error bars* indicate standard error of the mean. *Asterisks* indicate significant differences within an event (*Single asterisk* (*) for event AZ-4SX17; *two asterisks* for, event AZ-4SX28; *P* < 0.001) based on *t* tests. *No asterisks* means no significant difference within an event (events AZ-4SX37 and AZ-4SX16)

The OxO expression levels shown in Fig. 1 were generated from tissue culture shoots, whereas the leaves used in the small leaf assay were from potted plants. To determine whether the OxO expression levels in tissue culture shoots could predict expression in whole plants, RNA was extracted from small young stems of potted plants and analysed by RT-qPCR. Figure 2 shows four events in the 35S-OxO lines representing a range of tissue culture OxO expression levels and the corresponding expression levels in the stems of potted plants. The trend of OxO expression levels apparently shows a positive correlation between the tissue culture shoots and the small young stems. However, specific expression levels between shoots and stems from the same events were significantly different from each other, sometimes as much as two fold.

### *Cryphonectria parasitica* necrosis in small leaf assays

**Fig. 3:**
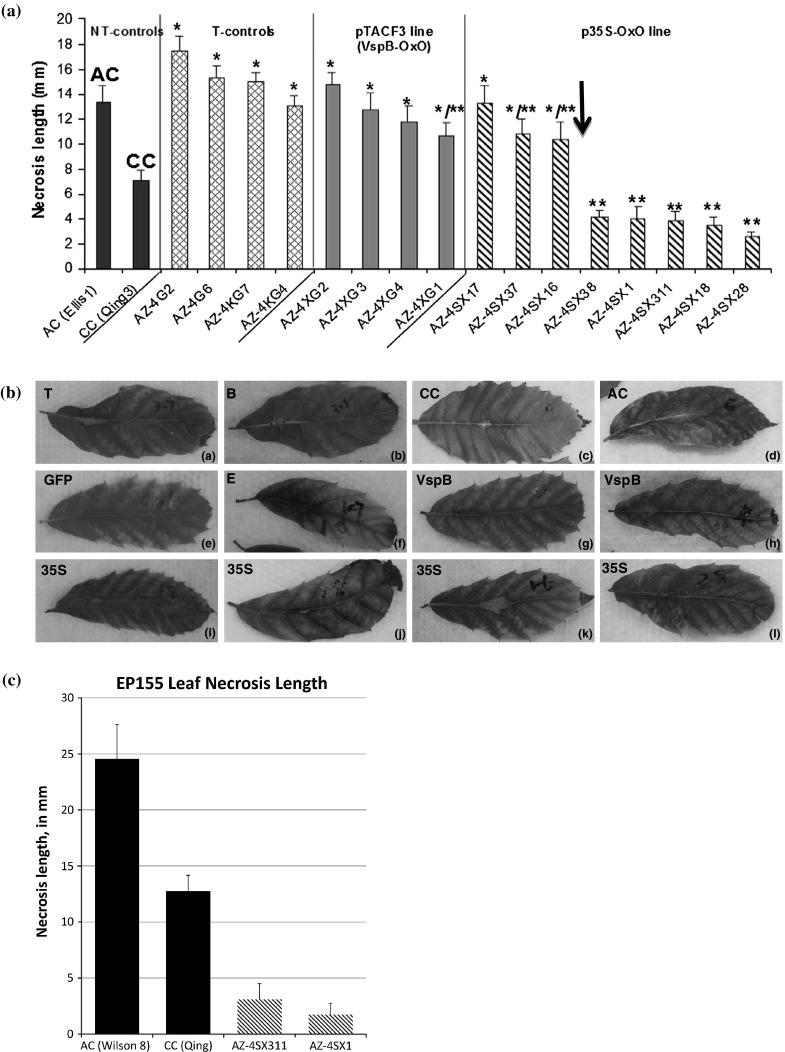
**a** Necrotic lesion length measurements from small leaf assays on mature leaves. Non-transgenic (*NT*) controls were the, American chestnut (*AC*, Ellis1,) for the blight susceptible standard and Chinese chestnut (*CC*, Qing3) for the blight resistant standard. Transgenic American chestnut controls (T-controls) without the OxO gene were AZ-4G2 and 6, (containing only the pGFP and; AZ-4KG4 and 7, containing the pWVK147 empty vector and pGFP. The pTACF3 transgenic events (containing the OxO with the vascular promoter) were AZ-4XG1, 2, 3, and 4. The p35S-OxO transgenic events (containing the constitutively expressed OxO) were AZ-4SX1, 16, 17, 18, 28, 37, 38, and 311. The order on the graph was arranged from he most necrosis to the least necrosis within each group. All plants were developed through tissue culture and grown in growth chambers. For each line/event, six to thirteen small mature leaves from different plants were used in the assay and lesion length along the midvein on the *upper side* of each leaf was measured in mm. *Bars* indicate standard error of the mean. AC and CC were used as non-transgenic controls and the standards; everything else was compared to them. A *single asterisk* (*) means *no* significant difference from the blight-susceptible American chestnut; *two asterisks* (**) mean *no* significant difference from the blight-resistant Chinese chestnut. ANOVA and a series of *t* tests were performed to determine significant difference (*P* < 0.005). (*AC* American chestnut, *CC* Chinese chestnut, *NT* non-transgenic, *T* transgenic) **b** Representative small leaf assay results. *T* top or adaxial side of the leaf; *B* bottom or abaxial side of the leaf with the *C. parasitica* inoculum SG2. Strain SG2 is of medium virulence and is tested to give significantly different lesion length between the Chinese and American chestnuts (Andrew Newhouse, unpublished); *CC* Chinese chestnut, *AC* American chestnut, *GFP* the pGFP line, *E* the empty vector pWVK147 line (pWVK147 has the exact same vector backbone as the *VspB*-OxO and the 35S-OxO lines, but without the promoter-gene cassette), *VspB* the pTACF3 (*VspB*-OxO) line, *35S* the p35S-OxO line. **a** AZ-4SX37 (*top* adaxial side); **b** AZ-4SX37 (*bottom* abaxial side), **c** CC (Qing3), **d** AC (Ellis1), **e** AZ-4G6, **f** AZ-4KG7, **g** AZ-4XG1, **h** AZ-4XG4, **i** AZ-SX18, **j** AZ-4SX38, **k** AZ-4SX16, **l** AZ-4SX28. *Arrow* indicates threshold OxO expression level (Fig. 1) linked to significant reduction in necrosis

To better understand the relationship between OxO expression and the blight fungus *in planta*, leaf assays were conducted using excised leaves from potted plants (Fig. 3). In this method, necrotic lesion length on the leaf midveins mimics relative blight resistance levels in susceptible American and resistant Chinese chestnut (Newhouse, unpublished). In the transgenic control lines without *oxo* (AZ-4G2, AZ-4G6, AZ-4KG7, AZ-4KG4), mean lesion length produced by *C. parasitica* strain SG2-3 among different events was not significantly different from blight-susceptible American chestnut (Fig. 3a). Also, none of the four events in the *VspB*-OxO line (AZ-4XG1 through AZ-4XG4) showed a significant difference from the blight-susceptible American chestnut (Fig. 3a). One of the *VspB*-OxO events (AZ-4XG1) also grouped statistically with the blight-resistant Chinese chestnut, which might indicate an intermediate level of resistance to fungus-induced necrosis. More interestingly, five events from the 35S-OxO line (AZ-4SX38, AZ-4SX1, AZ-4SX311, AZ-4SX18, and AZ-4SX28) showed significant mean necrotic lesion length differences compared to the American chestnut and no significant difference from the Chinese chestnut (Fig. 3a, last five events). These five 35S-OxO events fit into the same lesion length category as the blight-resistant Chinese chestnut, showing significantly reduced necrosis compared to their non-OxO counterparts. One other 35S-OxO event (AZ-4SX17) grouped with the American chestnut control, and two events (AZ-4SX37, AZ-4SX16) appeared to be intermediate, grouping with both the Chinese and American controls.

To confirm that the leaf assay results were not limited to the *C. parasitica* strain SG2-3, they were repeated on AZ-4SX1 and AZ-4SX311 with using strain EP155 (Fig. 3c). Both showed reduced lesion necrosis, this time even lower than the Chinese chestnut control.

### OxO expression level and lesion length

To explore the correlation between the OxO expression level and lesion length, the leaf assay results (Fig. 3a) were compared to the OxO transcript levels (Fig. 1a). The non-transgenic blight-susceptible American chestnut and the blight-resistant Chinese chestnut were used as the controls and the standards for blight susceptibility. The corresponding necrotic lesion length data were not clearly inversely proportional to OxO expression. However, when the expression of the OxO reached a certain level, or threshold (arrow in Fig. 1a), the necrosis was consistently reduced to the same level as the blight-resistant Chinese chestnut (arrow in Fig. 2a). This indicates that there is a threshold response between the OxO expression level and the *C. parasitica* lesion length.

**Fig. 4:**
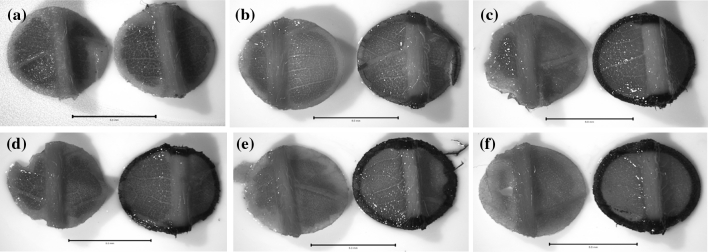
Oxalate oxidase (OxO) leaf disk assays. **a** Ellis 1 non-transgenic control, **b** AZ-4SX38, **c** AZ-4SX28, **d** AZ-4SX1, **e** AZ-4SX311, **f** AZ-4SX18. *Left leaf disks* no OA, *right leaf disks* added OA. *Dark* staining indicates a positive OxO reaction. *Bars* = 5 mm

Oxalate oxidase enzymatic activity was confirmed in leaf assays from regenerated whole plants (Fig. 4) for the five events (4SX38, 4SX1, 4SX311, 4SX18, and 4SX28) that showed the smallest necrotic lesions. The non-transgenic control (Ellis 1) showed no OxO activity.

## Discussion

We tested twenty-four transgenic events of the heritage tree American chestnut (Maynard et al. 2009), using vectors containing the oxalate oxidase gene from wheat, under the control of two different promoters, and empty vector controls. The transgenic events gave a wide range of *oxo* expression, allowing us to study the effects of OxO expression on *C. parasitica*’s ability to cause necrosis. The range of OxO expression levels observed in the 35S-OxO lines was most likely due to positional effect, in which the expression of the transgene is affected by the insertion site and nearby regulatory sequences. Similar results were observed in transgenic apple trees expressing the *uidA* gene (GUS) under the same CaMV 35S promoter (Gittins et al. 2001). The notable difference in the OxO expression levels between the 35S-OxO lines (high expression) and the *VspB*-OxO lines (low expression) was most likely due to the difference in the strength of the two promoters, the tissue specificity of the *VspB* promoter, and positional effect. Similar results were observed in transgenic peanut expressing GUS under the 35S and the *VspB* promoters (Wang et al. 1998). OxO has been shown to enhance fungal resistance in many transgenic plants (Chiriboga 1966; Lane 1994, 2002; Lane et al. 1993). Transgenic oilseed rape, tomato, sunflower, peanut, and soybean, all expressing *oxo* genes from wheat or barley, showed enhanced resistance and reduced disease symptoms to various different fungal pathogens including the OA-secreting fungi *Sclerotinia sclerotiorum*, *Botrytis cinerea* and *Sclerotinia minor* (Donaldson et al. 2001; Dong et al. 2008; Hu et al. 2003; Livingstone et al. 2005; Walz et al. 2008; Zou et al. 2007). OxO has also been shown to enhance resistance to *Septoria* in transgenic poplar (Liang et al. 2001). Moreover, OA-treated transgenic American chestnut callus tissue expressing a wheat OxO protected lignin (which impedes pathogen infection) from degradation (Welch et al. 2007).

A significant finding was that OxO expression correlated with *C. parasitica* necrotic lesion length in a threshold response manner (Figs. 1, 3 arrows). Although OxO was expressed in all tested events, all four *VspB*-OxO events and three 35S-OxO events showed lesion lengths that were statistically similar to the blight-susceptible American chestnut. Three of these also grouped with the Chinese chestnut, which might indicate an intermediate level of resistance in these events. The fact that a significant reduction in lesion length was only achieved when the level of the OxO expression reached a certain threshold (Figs. 1, 3 arrows) might suggest multiple resistance mechanisms because it is hypothesized that simply removing oxalic acid’s toxic effects would result in a more gradual dose response. OxO can degrade OA to carbon dioxide and hydrogen peroxide (Berna and Bernier 1999), thus neutralizing the toxic effects of the OA (Havir and Anagnostakis 1983). OA is one of the strongest organic acids, and can decrease the pH of woody tissues from 5.5 to 2.8, a pH level toxic to the plants but optimum for fungal enzymes (Dutton and Evans 1996; Jordan et al. 1996; Lane 1994; McCarroll and Thor 1978). Oxalate also has the ability to bind to divalent cations (i.e. calcium) in the cell wall, causing structural weakness (Dutton and Evans 1996; Havir and Anagnostakis 1983; Jordan et al. 1996; Lane 1994; McCarroll and Thor 1978). The sequestration of calcium by the oxalate works synergistically with the fungal enzyme polygalacturonase to weaken the plant cell walls, facilitating penetration of fungal hyphae (Dutton and Evans 1996). OA is also known to induce programmed cell death in some pathogen/host systems, which benefits necrotrophic pathogens (Errakhi et al. 2008; Kim et al. 2008) like *C. parasitica*. Oxalate is also involved in the regulation of fungal pathogenesis systems through pH signalling (Manteau et al. 2003; Rollins and Dickman 2001). OA (or oxalate) is a virulence factor secreted by many pathogenic fungi including *Botrytis cinerea*, *Sclerotinia sclerotiorum*, and the chestnut blight fungus *C. parasitica* (Dutton and Evans 1996). Without the production of oxalate, *C. parasitica* forms morphologically normal colonies on Potato Dextrose Agar, but fails to form a lethal canker upon inoculation (Chen et al. 2010). OA may also aid in the process of *C. parasitica* hyphae penetration and mycelial fan progression (Hebard et al. 1984). By degrading the OA secreted by *C. parasitica*, the speed of mycelial fan progression could be reduced, thus providing the transgenic trees more time to form a complete wound periderm to wall off the blight fungus and prevent further disease development.

Events from all four transgenic control lines (GFP-only and empty vector pWVK147) showed no significant difference in lesion length compared to the blight-susceptible American chestnut. This was expected since none of these events have putative resistance enhancing genes. One 35S-OxO event (AZ-4SX311) with a high OxO expression level exhibited reduced lesion length and contained a single copy of the transgene *oxo* (Zhang et al. 2011). Events containing a single transgene copy are highly desirable because oxo expression is stable.

Comparisons between OxO expression in shoots and greenhouse plants showed that the overall trend of the OxO expression levels in whole plants could be approximately predicted from the levels in tissue culture shoots (Fig. 2). However, for some events, the exact value between the tissue culture shoots and stems was shown to be statistically different for the same event, which could be caused by the positional effect. When the insertion site of the *oxo* gene is close to regulatory sequences that could be affected by developmental stages and different environmental factors, *oxo* transcription could be affected, resulting in significantly different expression levels between tissue culture shoots and stems. Therefore, variation in transgene expression is not unusual as plants mature and environmental conditions change. These differences were relatively small, with the highest showing only a two-fold change from tissue culture to potted plant. Therefore, precise levels of whole-plant transgene expression should not be extrapolated from analysis of tissue culture shoots, but general trends of transgene expression levels in whole plants may be predictable based on analysis of tissue culture shoots.

In summary, several events of two transgenic American chestnut lines (35S- and *VspB*-OxO) expressing a wheat *oxo* gene were successfully generated and compared. Desirable OxO expression levels were achieved in five 35S-OxO events, which also showed *C. parasitica* leaf lesion lengths similar to those found on leaves of blight-resistant Chinese chestnut.
